# A Comparative Study of Three Interneuron Types in the Rat Spinal Cord

**DOI:** 10.1371/journal.pone.0162969

**Published:** 2016-09-22

**Authors:** Si Chen, Guangqi Yang, Yaxi Zhu, Zongwei Liu, Weiping Wang, Jiayou Wei, Keyi Li, Jiajia Wu, Zhi Chen, Youlan Li, Shuhua Mu, Lisi OuYang, Wanlong Lei

**Affiliations:** 1 Department of Anatomy, Zhongshan School of Medicine, Sun Yat-sen University, Guangzhou, Guangdong, China; 2 Guangdong Province Key Laboratory of Brain Function and Disease, Zhongshan School of Medicine, Sun Yat-sen University, Guangzhou, Guangdong, China; 3 Department of Radiology, the First Affiliated Hospital, Sun Yat-Sen University, Guangzhou, Guangdong, China; 4 School of Medicine, Shenzhen University, Shenzhen, Guangdong, China; University of North Dakota, UNITED STATES

## Abstract

Interneurons are involved in the physiological function and the pathomechanism of the spinal cord. Present study aimed to examine and compare the characteristics of Cr+, Calb+ and Parv+ interneurons in morphology and distribution by using immunhistochemical and Western blot techniques. Results showed that 1) Cr-Calb presented a higher co-existence rate than that of Cr-Parv, and both of them were higher in the ventral horn than in the dosal horn; 2) Cr+, Calb+ and Parv+ neurons distributing zonally in the superficial dosal horn were small-sized. Parv+ neuronswere the largest, and Cr+ and Calb+ neurons were higher density among them. In the deep dorsal horn, Parv+ neurons were mainly located in nucleus thoracicus and the remaining scatteredly distributed. Cr+ neuronal size was the largest, and Calb+ neurons were the least among three interneuron types; 3) Cr+, Calb+ and Parv+ neurons of ventral horns displayed polygonal, round and fusiform, and Cr+ and Parv+ neurons were mainly distributed in the deep layer, but Calb+ neurons mainly in the superficial layer. Cr+ neurons were the largest, and distributed more in ventral horns than in dorsal horns; 4) in the dorsal horn of lumbar cords, Calb protein levels was the highest, but Parv protein level in ventral horns was the highest among the three protein types. Present results suggested that the morphological characteristics of three interneuron types imply their physiological function and pathomechanism relevance.

## Introduction

Because of neuron types, histological structure and neural circles, the dorsal horn, the intermediate gray and the ventral horn of the spinal cord show different physiological and pathological features. The dorsal horn mainly receives and processes the sensory information from dorsal root ganglion, and links between different spinal segments, and the neurons located in the spinal intermediate gray function on mediating the visceral activities [1, 2]. The neurons distributed in the ventral horn are mainly two subtypes: the large size called α neurons and the small γ neurons, which mediate skeletal muscle action [3]. The territorial function of the spinal cord is related to the corresponding parts of body; the action of upper limbs is mainly regulated by the cervical cord, and lower limbs, lumbosacral enlargement [1].

Excepting for the sensory neurons of the dorsal horn and the motor neurons of the ventral horn, there are many medium sized interneurons in the gray matter of spinal cords, and most of them are known as RCs (Renshaw cells)with GABA (γ-aminobutyricacid) or GLY (glycine) neurotransmitters [4–10], which form complex reciprocal inhibitive circles, and they mediate and influence the physiological function and pathological response of spinal cords. Cr (Calretinin), Calb (Calbindin-D_28k_) and Parv (Parvalbumin) are members of the calcium-binding protein family, which are known as major components of interneurons of spinal cords [11]. They are not only involved in the buffering of free intracellular calcium, but also play an important role in maintaining the calcium homeostasis of gray matter neurons [12–14]. Our previous studies showed that calcium-binding protein interneurons distributing in other brain areas displayed different features in morphology, distribution and characteristic reaction in these pathological processes of MCAO (middle cerebral artery occlusion), HD (Huntington’s disease) and PD (Parkinson’s disease)[15–19]. Studies showed that the distribution and morphology of interneurons of spinal cords were closely related with animal species; which in the spinal cord of mongrel dogs, Calb neurons were predominant in the superficial dorsal horn and layer IX, but Parv neurons in layers III and IV [20]. Meanwhile, Cr positive neurons were abundant in layers V-VII, and Calb interneurons mainly in the layers I-III, but most of Parv neurons were located in the layers IV-VIII in cats [21]. Although Cr, Calb and Parv interneurons displayed various morphological and distribution pattern, co-existence of Cr, Calb and Parv landmark proteins was found and showed different ratio in the spinal distinct areas of animal different species [22, 23].

Overall, the present study aimed to verifying the characteristics of Cr+, Calb+ and Parv+ interneurons in morphology and distribution in normal adult rats. These morphological data are of important significance for further understanding the physiological function and pathological mechanism of the spinal cord and its interneurons.

## Materials and Methods

### Experimental animals

Twelve adult male Sprague-Dawley (SD) rats weighing 250–300g (obtained from the Center for Experimental Animals of Sun Yat-sen University) were used in present study. The animals were housed in a room under an even dark/light cycle, and were given free access to water and standard rat diets. All animal experiments strictly adhered to the Regulations for the Administration of Affairs Concerning Experimental Animals, which was the Chinese national guideline for animal experiment, issued in 1988. All procedures involving animals and their care in this study were approved by the Animal Care and Use Committee of Sun Yat-sen University (Permit Number: SCXK GUANGDONG 2011–0029). Six rats were used for immunohistochemistry study, and the remainders were used for Western blot.

### Tissue preparation and immunohistochemistry of single-labeling

The rats used in immunohistochemistry experiment were deeply anesthetized with sodium pentobarbital (50 mg/kg, i.p.) and perfused transcardially with 300 ml of 0.9% sodium chloride followed by 400 ml of 4% paraformaldehyde (in 0.1 M phosphate buffer, pH 7.4). We chose the expend of the cervical cord (C6, C7, C8) and lumbar cord (L3, L4, L5). In addition, when referred to thoracic cord, we chose T6, T7 and T8. Based on previous studies, they presented the typical morphological structure of cervical cord, thoracic cord and lumbar cord of rat.[24–27]. Spinal cords were carefully removed, post-fixed in the same fixative overnight at 4°C. And then dehydrated in gradient sucrose solution (in 0.1 M phosphate buffer, pH 7.4). Sectioned (30 μm) on a semiconductor freezing microtome.

Sections were treated with 0.3% H_2_O_2_ in 0.1 M phosphate buffered saline (PB, pH 7.4) at room temperature for 30 min before incubated at 4°C for 36–40 hrs with one of the following primary anti-bodies: rabbit anti-Cr (1:2,000, catalog AB5054, Millipore), mouse anti-Calb (1:1,000, catalog SAB4200543, Sigma-Aldrich), mouse anti-Parv (1:5,000,catalog P3088, Sigma-Aldrich)diluted with 0.1 M PB, pH 7.4, containing 0.5% (w/v) BSA and 0.3% (v/v) Triton X-100 (PB-ST). Sections were rinsed and incubated in anti-rabbit IgG or anti-mouse IgG (both 1:100, Sigma-Aldrich) diluted with the same buffer mentioned above for 3 hrs at room temperature, followed by incubation in homologous PAP complex (1:200, Sigma) at room temperature for 2 hrs. The peroxidase reaction was performed using DAB (3, 3’-diaminobenzidine, 0.05% in 0.1 M PB, pH 7.4, Sigma) for 2–10 min. For each procedure, sections were repeatedly rinsed three times in 0.1 M PB, and each time for 5 min. Sections were mounted onto gelatin-coated slides, dehydrated, cleared with xylene, and covered with neutral balsam.

### Immunohistochemistry of double-labeling

In order to detect the density of interneurons, double-labeling immunohistochemistry was implemented as following pairs: Cr-NeuN, Calb-NeuN and Parv-NeuN, and immunohistochemistry procedures were the same as used above. Three interneuron types were immunolabeled, visualized with DAB solution containing 0.04% nickel ammonium sulfate (black), and then double-labeled with NeuN (mouse anti-NeuN, 1:1,000, catalog MAB377, Millipore), respectively. After finishing incubation for the secondary antibody and homologous PAP complex, the sections were visualized with DAB solution (brown).

Double-labeling of immunofluorescence was applied to detect and compare the co-existence relationship between Cr with Calb or Parv antigens. Following the above-mentioned immunohistochemistry procedures, i.e., the incubation of the first antibody (Cr), the sections were incubated with the secondary antibody (AF594, 1:400, donkey anti-rabbit, Invitrogen). For the double-labeling pairs of: Cr-Parv and Cr-Calb, following the incubation of the 1st antibodies (Calb and Parv), the sections were incubated with the secondary antibody (FITC, 1:400, goat anti-mouse, Chemicon). Finally, sections were mounted on gelatin-coated slides, and covered with glycerol. Section detection and image capture were conducted on a fluorescence microscope.

### Western blotting

Western blotting was carried out to detect the levels of marker proteins for each examined projection neuron type. All the rats were killed by decapitation after being deeply anesthetized with sodium pentobarbital (50 mg/kg, i.p.), and the spinal cords were extracted and homogenized in a RIPA buffer, to which protease inhibitors had been freshly added. The homogenate was centrifuged at 2,5000 rpm for 25 min, and the protein concentration of the homogenate was determined using the BioRad DC protein assay (BioRad Laboratories). 40μg of total protein from each sample were subjected to an SDS–PAGE (10%) and transferred to a PVDF membrane (Millipore). Membranes were blocked in 5% skim milk, and incubated with rabbit anti-Cr (1:8,000, catalog AB5054, Millipore), mouse anti-Calb (1:1,000, catalog SAB4200543, Sigma-Aldrich), mouse anti-Parv (1:3,000, catalog P3088, Sigma-Aldrich) or rabbit anti-GAPDH (1:2,000, Cell Signaling Technology) overnight at 4°C. Incubated membranes were then treated with secondary antibody conjugated with horseradwash peroxidase (1:3,000, Chemicon) for 2 hrs at 37°C.

### Data collection and statistical analysis

We had divided the gray matter to three regions: the superficial dorsal horn, the deep dorsal horn and the ventral horn. Previously [14,60,67], the superficial dorsal horn (laminae I and II) appears as a distinctive dark band under these conditions, due to the relative lack of fibres [28]. So, we distinguished the bundary of deeper dorsal horn and superficial dorsal horn. The position of the lamina III-IV border was determined from previous research.[24]

For quantitative analysis by light microscope (Olympus), the cell counting of Cr+, Calb+ and Parv+ interneurons were determined according to the following procedure: each sampled section was firstly viewed at 100× magnification with a reticule (0.1mm×0.1mm) in one eyepiece to observe the whole area of grey matter of the spinal cord, and then the reticule was randomly moved into five non-overlapping regions (0.01mm^2^ for each regions) within the gray matter. The somas size was measured at 400× magnification with a 100 μm reticule. The soma size was the average of the major axis and minor axis.

Blots were developed by enhanced chemilu-minescence and digitally scanned using ImageQuant Las4000mini. The optical density of Cr, Calb, and Parv labeled band was normalized against their respective GAPDH by using Image-Pro Plus 6.0 software.

All data in this study are presented as means ± SD. The statistical significance of the results was evaluated by one-way ANOVA or Student’s t test using SPSS analytical software 16.0, and p < 0.05 was considered as significant. The details for counting and measuring the neurons aboved were according to our previous methods [15, 17, 18, 29].

## Result

### 1 The comparison of co-existence ratio between Cr and Calb or Parv antigens in spinal cords

**Fig 1:**
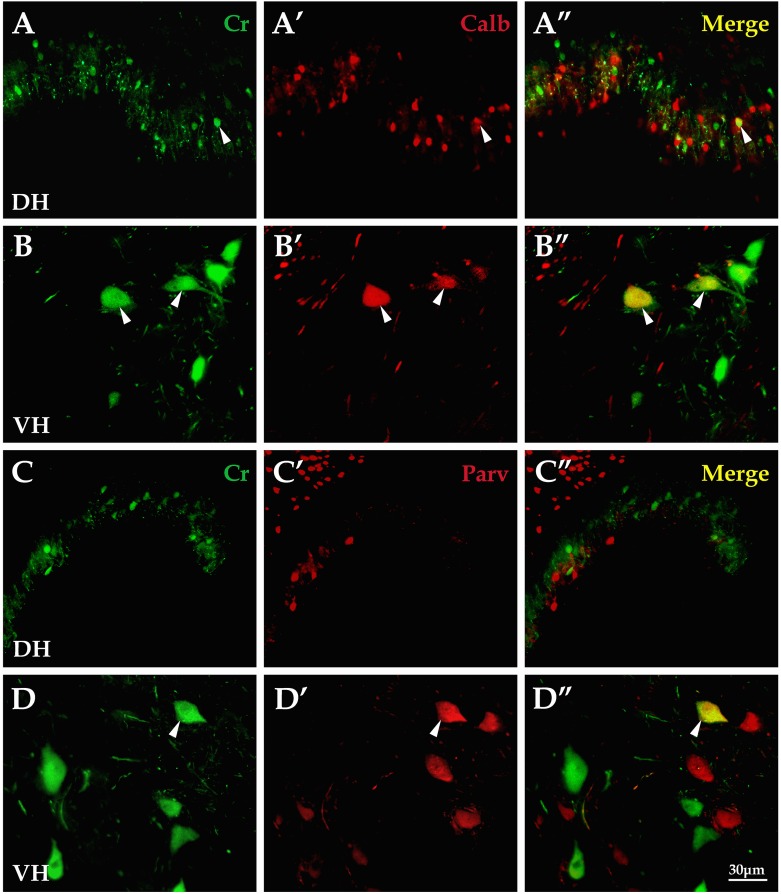
The detection of co-existence relation for Cr with Calb and Parv in the spinal cord. Layers I-II(A-A″) and ventral horn (B-B″),respectively, showed Cr (green), Calb (red), and Cr-Calb co-existence neurons (yellow). Layers I-II (C-C″) and the ventral horn(D-D″) respectively, showed Cr (green), Parv (red), and Cr-Parv co-existence neurons (yellow).Cr, Calb and Parv are short for Calretinin, Calbindin-D_28k_, Parvalbumin, and DH and VH are short for the dorsal horn and the ventral horn, respectively. All images are the same magnification.Scale bar: 30μm.

Immunofluorescence double-labeling was applied to detect and compare the co-existence relation between Cr and Calb or Parv antigens. Results showed that the double-labeling percentage(%) for Cr with Calb neurons (14.6±2.3) was higher than that of Cr-Parv (10.9±1.8; *P*<0.05), and the co-existence ratio for Cr-Calb were higher in both the dorsal horn (6.8±1.2, Fig 1A–1A″) and the ventral horn (22.5±1.1, Fig 1B–1B″) than that of Cr-Parv(4.1±1.1; 16.7±1.2; *P*<0.05; *P*<0.05, Fig 1C–1C″ and 1D–1D″). Compared between the dorsal horn and the ventral horn, the double-labeling percentages of Cr-Calb (6.8±1.2: 22.5±1.1; *P*<0.05) and Cr-Parv (4.1±1.1: 16.7±1.2; *P*<0.05) were significantly different (Fig 1; Table 1).

**Table 1:** The co-existence of Cr with Calb or Parv antigens.

	Total(%)	DH(%)	VH(%)	*P*
Cr-Calb	14.6±2.3	6.8±1.2	22.5±1.1*	<0.05
Cr-Parv	10.9±1.8^#^	4.1±1.1^#^	16.7±1.2*^#^	<0.05
*P*	<0.05	<0.05	<0.05	

Note;

*P<0.05, compared between DH and VH;

^#^*P*<0.05, compared between Cr-Calb and Cr-Parv. Cr, Calb and Parv were short for Calretinin, Calbindin-D_28k_, Parvalbumin, and DH and VH were short for the dorsal horn and the ventral horn, respectively.

### 2 The morphological comparison of Cr+, Parv+, Calb+ interneurons of spinal cords

#### The dorsal horn of spinal cords

**Fig 2:**
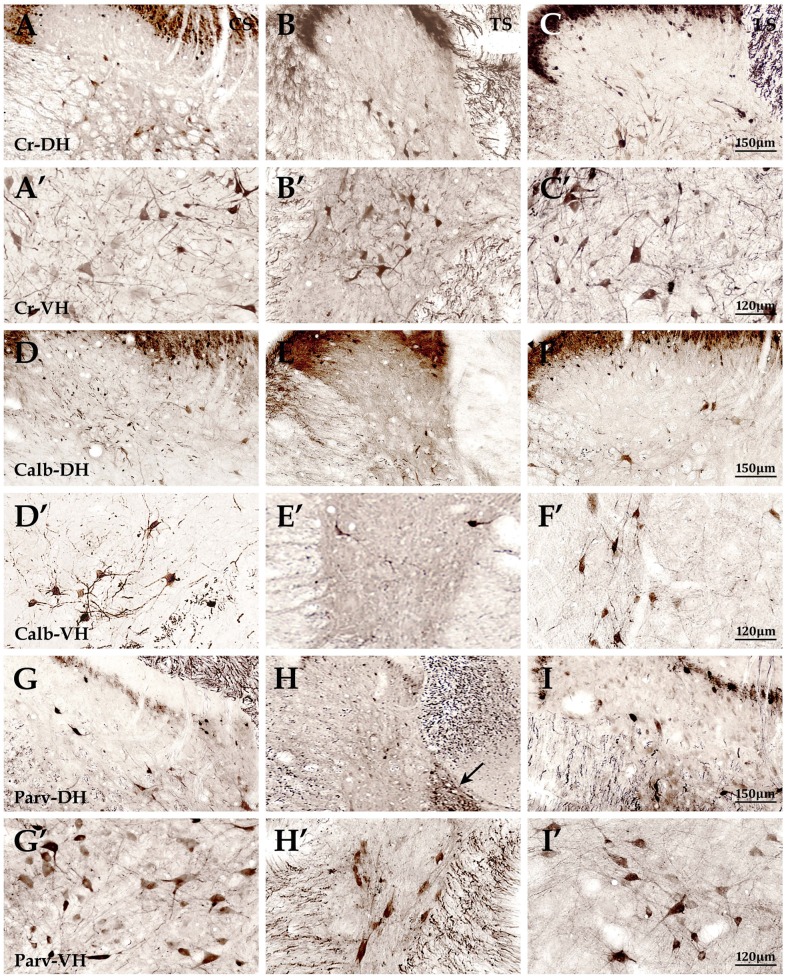
The morphological exploration of interneurons of the spinal gray matter. Figures (A-C) showed Cr+ neurons in the dorsal horns of cervical (A), thoracic (B) and lumbar cord (C),and A′- C′ showed Cr+ neurons in the ventral horns of cervical (A′), thoracic (B′) and lumbar cord (C′), respectively.Figures (D-F) showed Calb+ neurons in the dorsal horns of cervical (D), thoracic (E) and lumbar cord (F),and Figures (D′- F)showed Calb+ neurons in the ventral horns of cervical (D′), thoracic (E′) and lumbar cord (F′), respectively.Figures G, H and I showed Parv+ neurons in the dorsal horns of cervical (G), thoracic (H) and lumbar cord (I), and the arrows showed the nucleus thoracicus which Parv+ neurons gathered (Fig.H). Figures (G′- I′) showed Parv+ neurons in the ventral horns of cervical (G′), thoracic (H′) and lumbar cord (I′), respectively. Cr, Calb and Parv are short for Calretinin, Calbindin-D_28k_, Parvalbumin, and CS, TS and LS are short for the cervical, thoracic and lumbar cords, respectively, and DH and VH are short for the dorsal horn and the ventral horn. The figures of dorsal horns are the same magnification. Scale bars: 150μm. The figures of ventral horns are the same magnification. Scale bars: 120μm.

**Fig 3:**
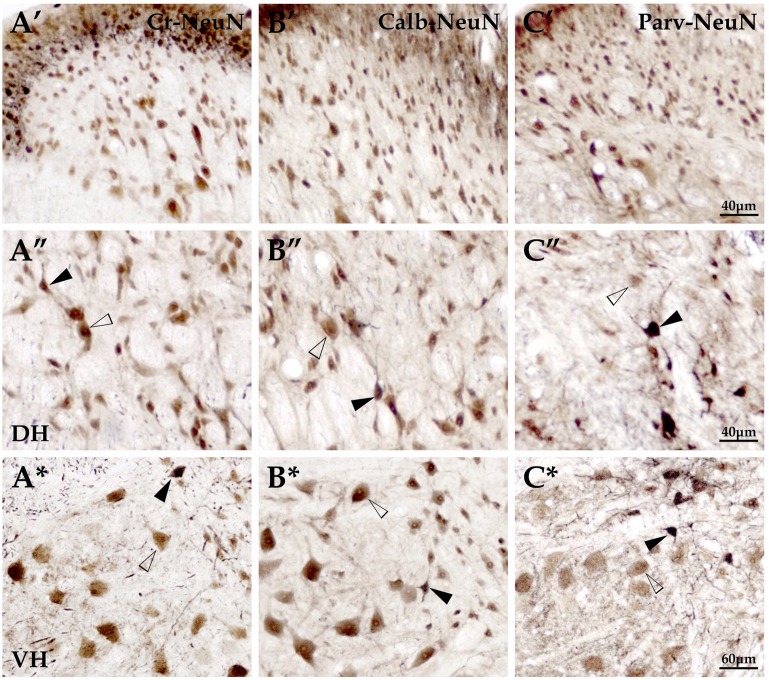
The double-labeling for interneurons with NeuN+ neurons of the spinal gray matter. The double-labeling for Cr-NeuN (A′-A*), Calb-NeuN (B′-B*) and Parv-NeuN (C′-C*) were showed above. Panels A′- C′, A″-C″ and A*-C* showed the superficial layer and the deep layer of dorsal horn as well as the ventral horn, respectively. Double-labeling neurons(▲) and NeuN+ neurons(△) were obviously observed. DH and VH are short for the dorsal horn and the ventral horn. Scale bar: A′-C′, A″-C″, 40μm; A*-C*, 60μm.

The superficial dorsal horn (SDH) of the mammalian spinal cord, laminae I and II, is a major termination region for thin primary afferent fibers.[28] In the superficial dorsal horn, Cr+, Calb+ and Parv+ neurons showed tiny spherical and intensive zonal distribution (Figs 2A–2C, 2D–2F, 2G–2I and 3A′–3C′). Quantitative data showed that the size (μm) of Parv+ neurons (8.0±0.9) was the largest compared with both Cr+ (7.7±1.2; *P*<0.05) and Calb+ neurons (6.6+1.0; *P*<0.05; Figs 2A–2C, 2D–2F, 2G–2I and 3A′–3C′). Parv+ neurons of lumbar cord (9.6±1.6) were also the largest in comparison with cervical (6.8±1.6) and thoracic cords (7.7+1.0; *P*<0.05; 2G-I). The size of Cr+ and Calb+ neurons did not show any regional difference, but the mean density (number/ 0.01mm^2^) of Cr+ (5.3±0.5) and Calb+ neurons (4.5±0.4) was higher than that of Parv+ neurons in the superficial dorsal horn (2.7±0.4; *P*<0.05). In addition, their densities showed no differences among cervical, thoracic and lumbar cords (*P*>0.05, Fig 2A–2C and 2D–2F). However, Parv+ neurons of the lumbar cord (4.1±1.6) had a higher density than that of cervical (2.2±0.4) and thoracic cords (1.9±0.4; *P*<0.05; Fig 2G–2I).

**Fig 4:**
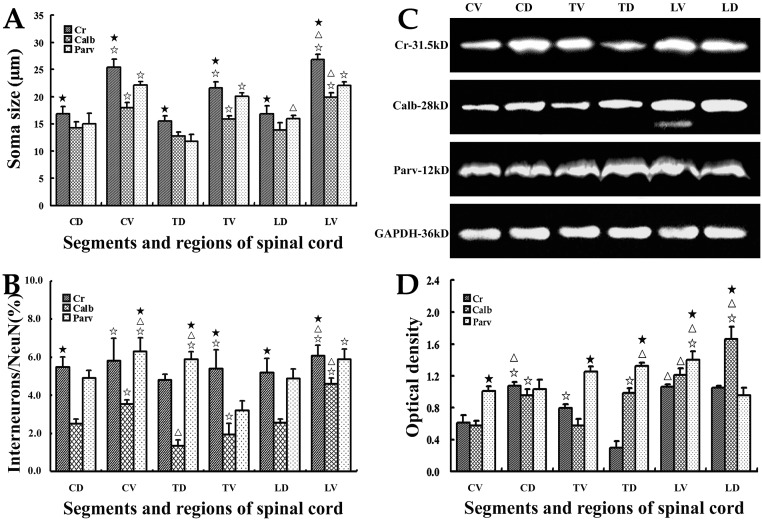
The comparison of Cr+, Calb+ and Parv+ neurons and their protein levels. The comparison result of the somas size of Cr+, Calb+ and Parv+ neurons in different segments and regions was showed (Histogram A), and histogram B showed the quantitative analyses of their density. HistogramC and D displayed the comparison of expression levels for Cr, Calb and Parv proteins. Values are expressed as group means±SD. ^☆^*P*<0.05, comparison between the dorsal horn and the ventral horn;^Δ^*P*<0.05, comparison among the cervical, thoracic and lumbar cords; ^★^*P*<0.05, comparison among three interneuron types in the same region of different spinal segments. DH and VH are short for dorsal horn and ventral horn, and CS, TS, LS are short for cervical, thoracic and lumbar cords.

In the deep dorsal horn, Cr+, Calb+ and Parv+ neurons presented polygonal, round and fusiform appearances(Figs 2A–2C, 2D–2F, 2G–2I and 3A′–3C′, 3A″–3C″). Statistical data showed that the size (μm) of Cr+ neurons (16.6±4.4) was larger compared with Calb+ (13.7±3.0) and Parv+ neurons (14.3±5.8; *P*<0.05), but each of them showed no regional differences among different spinal segments. Their size was slightly smaller in the thoracic cord than that in cervical and lumbar cords (Figs 2, 3A′–3C′ and 4A). Cr+ and Calb+ neurons distributed scatteredly in the deep layer of dorsal horn (layer III, 2A-C, D-F), while Parv+ neurons were mainly located at the nucleus thoracicus (layers V, VI; Fig 2G–2I). Double-labeling immunohistochemistry (interneuron/ NeuN) data showed that the double-labeling rate of Calb-NeuN (2.1±0.2) was markedly lower than that of Cr-NeuN (5.1±0.4) and Parv-NeuN (5.3±0.3; *P*<0.05; Fig 3A′–3C′ and 3A″–C″) and the percentages of Cr-NeuN and Calb-NeuN did not show obviously regional difference among cervical, thoracic and lumbar cords (Fig 4B), but Parv-NeuN had a higher rate in the thoracic cord than in cervical and lumbar cords (*P*<0.05; Fig 4B).

#### The ventral horn of spinal cords

Results showed that Cr+, Calb+ and Parv+ neurons in the ventral horn displayed polygonal, round and fusiform, and that the mean size (μm) of Calb+ neurons (18.3±4.0) was the smallest compared to Cr+ (24.6±5.0) and Parv+ neurons (22.5±3.1; *P*<0.05; Figs 2A′–2C′, 2D′–2F′, 2G′–2I′, 3A*–3C* and 4A). Cr+ (21.8±3.0), Calb+ (15.9±3.0) and Parv+ neurons (20.4±3.6) in thoracic cords were all smaller than that in their cervical and lumbar cords (all *P*<0.05; Fig 4A), and three positive neuron types were all larger in the ventral horn than in the dorsal horn (all *P*<0.05; Figs 2, 3 and 4A). The Cr+ and Parv+ neurons were mainly located in the deep layer of ventral horns, but Calb+ neurons mainly in the superficial layer (Fig 2). Among three interneuron types, double-labeling percentage (interneuron/NeuN) for Cr-NeuN (5.8±0.7) were the highest compared to Calb-NeuN (3.4±0.7) and Parv-NeuN (5.1±0.4; *P*<0.05; Fig 3A*–3C*). However, double-labeling ratio for Cr-NeuN (5.4±0.7), Calb-NeuN (1.9±0.6) and Parv-NeuN (3.2±0.5) were lower in the thoracic cord than in both cervical (5.9±1.2, 3.5±0.2, 6.2±0.8) and lumbar cords (6.1±0.6, 4.7±0.3, 5.9±0.5; all *P*<0.05). Among three neuron types in the same region of spinal cords, the double-labeling percentages of Calb-NeuN were the lowest in ventral horns of different spinal segments (*P*<0.05, Fig 4B), but Parv-NeuN showed the highest double-labeling percentage in the ventral horn of cervical cords (*P*<0.05, Fig 4B) and Cr-NeuN was the highest in the ventral horn of thoracic and lumbar cords (*P*<0.05, Fig 4B).

### 3 The exploration and comparison for Cr, Calb, Parv protein level

Western blot was applied to further detect and compare the expression levels of Cr, Calb, and Parv proteins of spinal cords in the present study. The results showed that in the dorsal horn, Cr protein level (0.9±0.3) was the lowest compared to Calb (1.2±0.2) and Parv protein levels (1.1±0.1; *P*<0.05; Fig 4C and 4D). The expression level of Cr protein (0.3±0.1) was the lowest in thoracic cord compared to cervical (1.1±0.1) and lumbar cords (1.1±0.2; *P*<0.05; Fig 4C and 4D), and Parv protein (1.3±0.1) showed a higher level than in cervical (1.0±0.2) and lumbar cords (1.0±0.1; *P*<0.05; Fig 4C and 4D). However, Calb protein level of the lumbar cord (1.7±0.2) was the highest in the comparison with cervical (0.9±0.1) and thoracic cords (1.0±0.2; *P*<0.05; Fig 4C and 4D).

In the ventral horn, Parv protein level (1.2±0.3) was the highest compared to Cr (0.8±0.2) and Calb (0.7±0.1), and all the three protein types were up-expressed in the lumbar cords (1.1±0.1, 1.2±0.1, 1.4±0.3) compared to their cervical (0.6±0.1, 0.5±0.1, 1.0±0.1,all *P*<0.05; Fig 4) and thoracic cords (0.8±0.2, 0.6±0.1, 1.3±0.5,all *P*<0.05; Fig 4). In the dorsal horn and the ventral horn, both Cr and Parv protein levels showed no significant differences, but Calb protein level was markedly higher in the dorsal horn (1.2±0.2) than in the ventral horn (0.7±0.1; *P*<0.05; Fig 4C and 4D).

## Discussion

### The morphological characteristic of spinal cord interneurons

The territorial regulating function of the spinal cord is mainly dependent on its inherent neuronal property and architecture, which play their role by the neural circuits known as “central pattern generators”; the neurons in the dorsal horn mainly relay sensory information and control skeletal muscle action in the ventral horn. Moreover, they modulate the visceral activity [1, 2, 30] in the intermediate gray. The spinal gray matter contains a lot of inhibitory interneurons, among which Calb and Parv are well known as typical inhibitory interneurons and mediate their physiological function by GABA and glycine neurotransmitters [21]. However, Cr interneurons regulate the proprioception by glutamatergic neurotransmitter [11, 21].

Studies found that Cr, Calb and Parv interneurons showed different characteristics of morphology and distribution. Calb neurons in mongrel dogs were mainly located in the superficial dorsal horn, while Parv neurons were abundant in layers III and IV [20]. Studies in cat spinal cords found that Parv neurons were located in layers IV-VIII and Cr neurons mainly existed in layers V and VI. Calb neurons were found in layers I-III and IX [31]. Our present results from adult rats showed that Cr+, Calb+ and Parv+ interneurons located in the superficial dorsal horn displayed intensive zonal distribution, and in the deep dorsal horn, Cr+ and Calb+ neurons distributed scatteredly and Parv+ neurons were mainly located in nucleus thoracicus. Cr+ and Parv+ neurons were mainly located in the deep layer in the ventral horn, but Calb+ neurons in the superficial layer. Our results simultaneously showed that their morphology, density and distribution presented regional differences. Cr+ and Parv+ neurons in the cervical and lumbar cords which control the actions of up and low limbs were obviously larger and abundant. These results were consistent with the study from mice, showing that Cr+ neurons were larger and Calb+ neurons were the least abundant population, but Parv+ neurons were the highest in number [32]. Western Blot in previous studies and in our study showed that the abundance of Calb protein in the dorsal horn and Parv protein in the ventral horn had tight connection with their function, but they were not fully consistent with the immunohistochemistry data. Similar results were also found in the studies of primate spinal cords [13, 33, 34]. Despite of the various morphological characteristic of the Cr, Calb and Parv interneurons, their different co-existence ratio had been found in rats [23], mice [35], and cats [31], in which the co-expression of Calb and Parv was more abundant in the ventral horn than in the dorsal horn. It is noted that our present results also showed the difference of co-existence ratio between Cr-Calb and Cr-Parv, and the difference of co-existence ratio was also found from PW2 to mature in the developmental neuroscience [21].

### The functional relevance of spinal cord interneurons

Calb and Parv, formed reciprocal inhibition in motor pools and feedback pathway with motor neurons to control muscle action [32], [36], [37], and the interneurons in the dorsal horn regulated the pain information by the theory konwn as " the Gate Theory"[38, 39]. Calb+ and Parv+ in the superficial dorsal horn (SDH) might also participate in proprioceptive system of lesion process due to the important role of GABAergic and somatostatin. Previous studies also had explored the distribution of such inhibitory interneurons in rat, mice and lamprey [28, 40, 41]. Based on the columnar organization of cat spinal cords, Rexed classified it into ten layers that were associated with corresponding function, and found that the interneurons located in different layers showed distinct morphological and distributive characteristic [3, 42]. Our present and other studies from different animal species showed that Cr+ neurons were numerous in the layers VI and VII, which received proprioceptive afferents. This suggested that Cr interneurons might be involved in posture and movement regulation. The study of Celsr3|Emx1 mice model showed that Cr neurons in C3-C4 segments were significant increased, which indicated contributing to remodeling of spinal motor circuits [43]. Both others’ and our present data showed that Calb interneurons were located obviously in the superfical dorsal horn and layer IX of rat and mouse spinal cords [13, 32, 35]. However, Parv neurons of mongrel dogs were abundant in the nucleus thoracicus [20], in which neurons were confirmed to project cerebellum by nervous tracing study in mice [44]. Present results showed that numerous Parv+ interneurons were clearly located in this nucleus, which implied their function relevance with propriocetion.

Cr, Calb and Parv interneurons were important members of calcium-binding proteins (CBPs) family, which not only regulate the buffering of free intracellular calcium and maintain the calcium homeostasis of gray matter neurons [12–14], but are also involved in pathological processes of spinal cords. Recently, study of West Nile Virus (WNV) also discovered that the neurons containing no Calb proteins were more susceptible to WNV infection [45], and CBP interneurons could escape the neurodegenerative insult [46, 47]. Studies confirmed that Parv interneurons were sensitive to peripheral neural inflammation [34], and our previous studies showed that Cr, Calb and Parv interneurons in the rat striatum displayed pathologic hyperplasia in the models of 3-nitropropionic acid (3NP)[15], quinolinic acid (QA)[16], and deprivation of dopamine [17]. In summary, our present morphological data from rat spinal cords were significant for understanding of the physiological function and pathological mechanism of both the interneurons and the spinal cord itself.

## Supporting Information

S1 File The raw data in the text could be found in the supporting file(Excel2003 format). The file containsix tables. Table A in S1 File. The co-existence ratio between Cr with Calb or Parv antigens in spinal cords.Cr, Calb and Parv were short for Calretinin, Calbindin-D28k, Parvalbumin, respectively. DH and VH for the dorsal horn and the ventral horn. Each data were averaged from 9 raw data. Table B in S1 File. The size of Cr+, Calb+ and Parv+ neurons in CS,TS and LS (um).Cr, Calb and Parv were short for Calretinin, Calbindin-D28k, Parvalbumin, and CS, TS and LS were short for the cervical, thoracic and lumbar cords, respectively. DH and VH for the dorsal horn and the ventral horn. Each data were averaged from 9 raw data. Table C in S1 File. The densityof Cr+, Calb+ and Parv+ neurons in CS, TS and LS (%). Cr, Calb and Parv were short for Calretinin, Calbindin-D28k, Parvalbumin, and CS, TS and LS were short for the cervical, thoracic and lumbar cords, respectively. DH and VH for the dorsal horn and the ventral horn. Each data were averaged from 15 raw data. Table D in S1 File. The sizeof Cr+, Calb+ and Parv+ neurons in superfical dorsal horn of CS, TS and LS (um). Cr, Calb and Parv were short for Calretinin, Calbindin-D28k, Parvalbumin, and CS, TS and LS were short for the cervical, thoracic and lumbar cords, respectively. DH for the dorsal horn. Each data were averaged from 9 raw data. Table E in S1 File. The densityof Cr+, Calb+ and Parv+ neuronsin superfical dorsal horn of CS, TS and LS (/0.01mm^2^). Cr, Calb and Parv were short for Calretinin, Calbindin-D28k, Parvalbumin, and CS, TS and LS were short for the cervical, thoracic and lumbar cords, respectively. DH for the dorsal horn. Each data were averaged from 15 raw data. Table F in S1 File. The protein level of Cr, Calb and Parv of CS, TS and LS (/GAPDH).Cr, Calb and Parv were short for Calretinin, Calbindin-D28k, Parvalbumin, and CS, TS and LS were short for the cervical, thoracic and lumbar cords, respectively. DH and VH for the dorsal horn and the ventral horn. Each data were averaged from 3 raw data.
(XLSX)
